# Forensic Significance of Adipocere Formation in Various Scenarios: A Case Series

**DOI:** 10.7759/cureus.84283

**Published:** 2025-05-17

**Authors:** Aravindan U, Senthil Kumaran M, Rajesh R, Aravinth R, Manigandan G

**Affiliations:** 1 Forensic Medicine and Toxicology, All India Institute of Medical Sciences, Madurai, Madurai, IND; 2 Forensic Medicine, Indira Gandhi Medical College and Research Institute, Puducherry, IND; 3 Forensic Medicine, Government Medical College, Ramanathapuram, Ramanathapuram, IND

**Keywords:** adipocere, decomposition, identification, injuries, mutilation

## Abstract

Adipocere formation is observed mainly in drowned bodies or bodies stored in airtight conditions for an extended period, which show evidence of a partly wax-like or plastic condition. It becomes extremely difficult for crime investigators to determine the cause of death in bodies in an advanced stage of decomposition. In such instances, changes like the formation of adipocere and mummification help to preserve certain features and injuries that aid in the determination of identity and the cause of death of the body recovered. The time required for the formation of adipocere is a subject of controversy, with various factors playing a role. Here, we present three cases of adipocere formation, which helped in determining the identification and cause of death.

## Introduction

Taphonomy is defined as the study of postmortem decaying processes of the dead body and its remains from the time of death to the time of recovery [1]. The decaying of human remains is a process rather than an event. It starts shortly after death, and the changes usually occur sequentially with various stages, including autolysis and putrefaction, under certain environmental conditions [2]. The fate of a dead body is to either proceed with putrefaction until the soft tissues have liquified and the corpse is completely converted into a skeleton. Otherwise, the process of putrefaction must be arrested, and modification in the putrefactive changes can occur, i.e., adipocere formation or mummification in favorable environmental conditions [3].

Various external and internal environmental conditions majorly influence the degree of decomposition. The external factor includes atmospheric temperature, humidity, type of soil, pH, water content of the soil where the body is disposed, and the presence or absence of clothes covering the dead body, and the internal factors include age, sex of the deceased, built and size of the body, availability of fat tissues, cause of death, medical conditions altering the process of putrefaction, and open external wounds in the body [4,5]. In damp and aquatic surroundings, these external factors will differ and alter the process of decomposition; it may include the effects of underwater currents, temperature of the water, depth of immersion, natural aquatic, and microfauna [6].

Adipocere, the term coined by Antoine Francois Fourcroy in 1789, is derived from the words "adipo" and "cire" to indicate its meaning of fat and wax, respectively, during the removal of a vast number of deceased from the Cimetiere des Innocents in Paris [7]. Adipocere is a grayish-white, waxy, soap-like substance, produced from the decomposition of adipose tissue, which converts adipose tissue into triglycerides, fatty acids, and other products. Adipocere forms over the body's external surface and the fat-covered internal viscera with time [8]. The ideal environmental factors considered appropriate for the formation of adipocere are adequate adipose tissue, an anaerobic environment, warm temperatures, moisture either from the environment or from the body itself, alkaline pH of the soil, and involvement of anaerobic gram-positive bacteria such as Clostridium perfringens and Clostridium frigidicanes [9]. Adipocere formation is one of the most vital late postmortem changes, as it may be applied to estimate time since death, identification, and interpretation of wounds, as it preserves the body structure. Here, we describe three cases of adipocere formation with various backgrounds, which were helpful in forensic investigation in crime solving and helping law enforcement.

## Case presentation

Case 1

A dismembered corpus of a 75-year-old male, with an alleged history of missing for the past three days as per an inquest report, was received for autopsy in the mortuary of a tertiary care hospital in South India. Police interrogation with the deceased wife and son revealed that the deceased was murdered and his body was cut into multiple pieces, packed in a sack, and thrown in a waterlogged dump yard.

**Figure 1:**
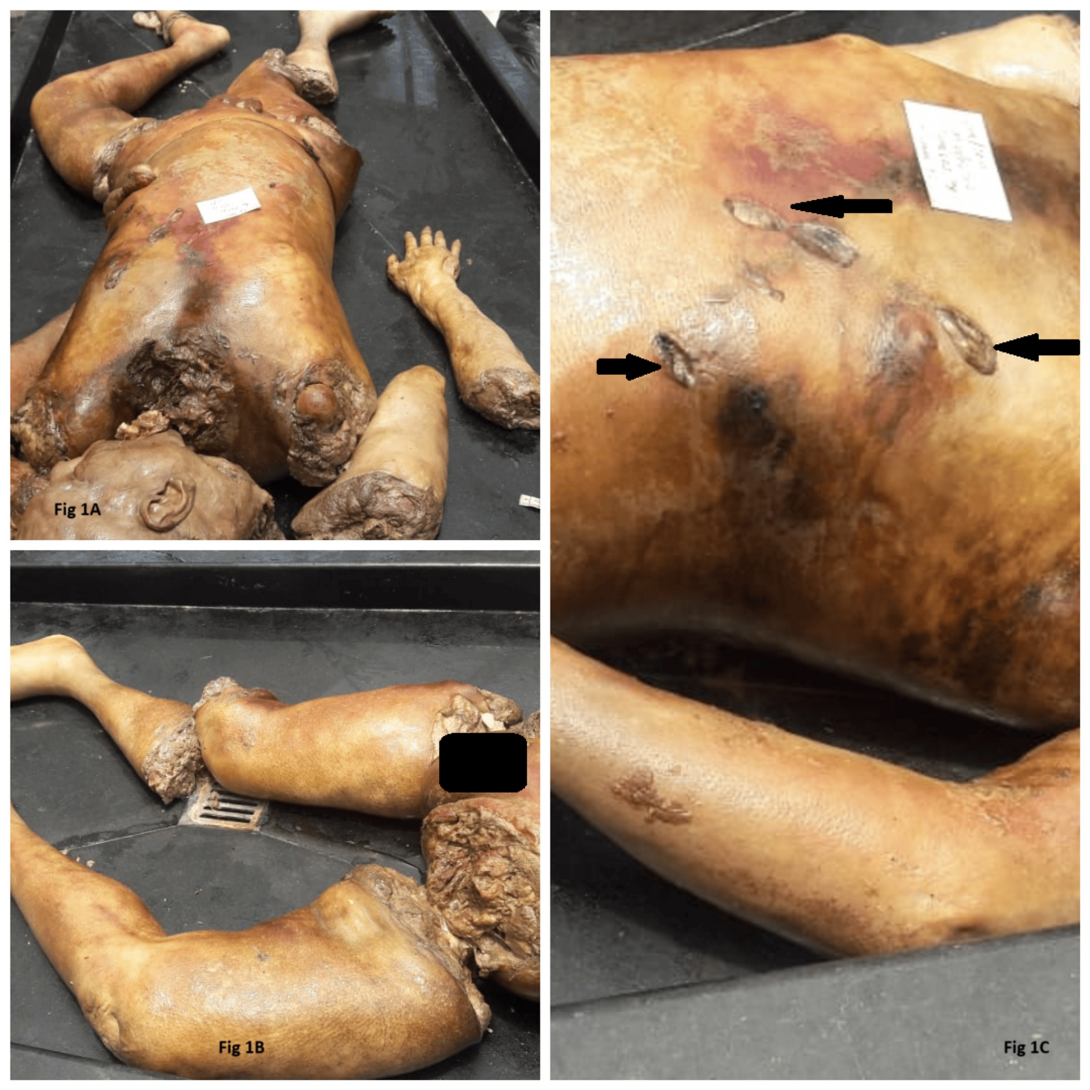
A) The mutilated and dismembered body parts arranged anatomically. B) The postmortem dismembered body parts of the lower limbs. C) Three stab wounds and their features are conserved due to adipocere formation on the left side of the anterior chest and abdominal wall.

On examination, the dead body was mutilated and chopped into eight pieces, separated as the right arm, right forearm with the right hand, left upper limb, decapitated head, torso, left lower limb, right thigh, and right leg with the right foot. The mutilated body parts were arranged based on complexion, contour, built, morphological structure, and anatomical alignment (Figure 1A, 1B).

It was a moderately built male body wearing no clothes and in a state of decomposition, with a whitish soft adipocere formation all over the body. Body parts were emitting a rancid, foul smell. The soft tissues at the cut ends were soft and greasy. On further examination, there were three stab wounds present on the left side of the chest and abdomen extending to the thoracic and abdominal cavity deep, which was of ante-mortem in nature (Figure 1C). All internal organs showed decomposition changes. Toxicological analysis of viscera ruled out poisoning and other intoxication. The cause of death was opined as hemorrhage and shock resulting from a stab injury to the chest and abdomen caused by a single-edged sharp cutting weapon having a pointed tip, and the time since death was two to three days.

Case 2

**Figure 2:**
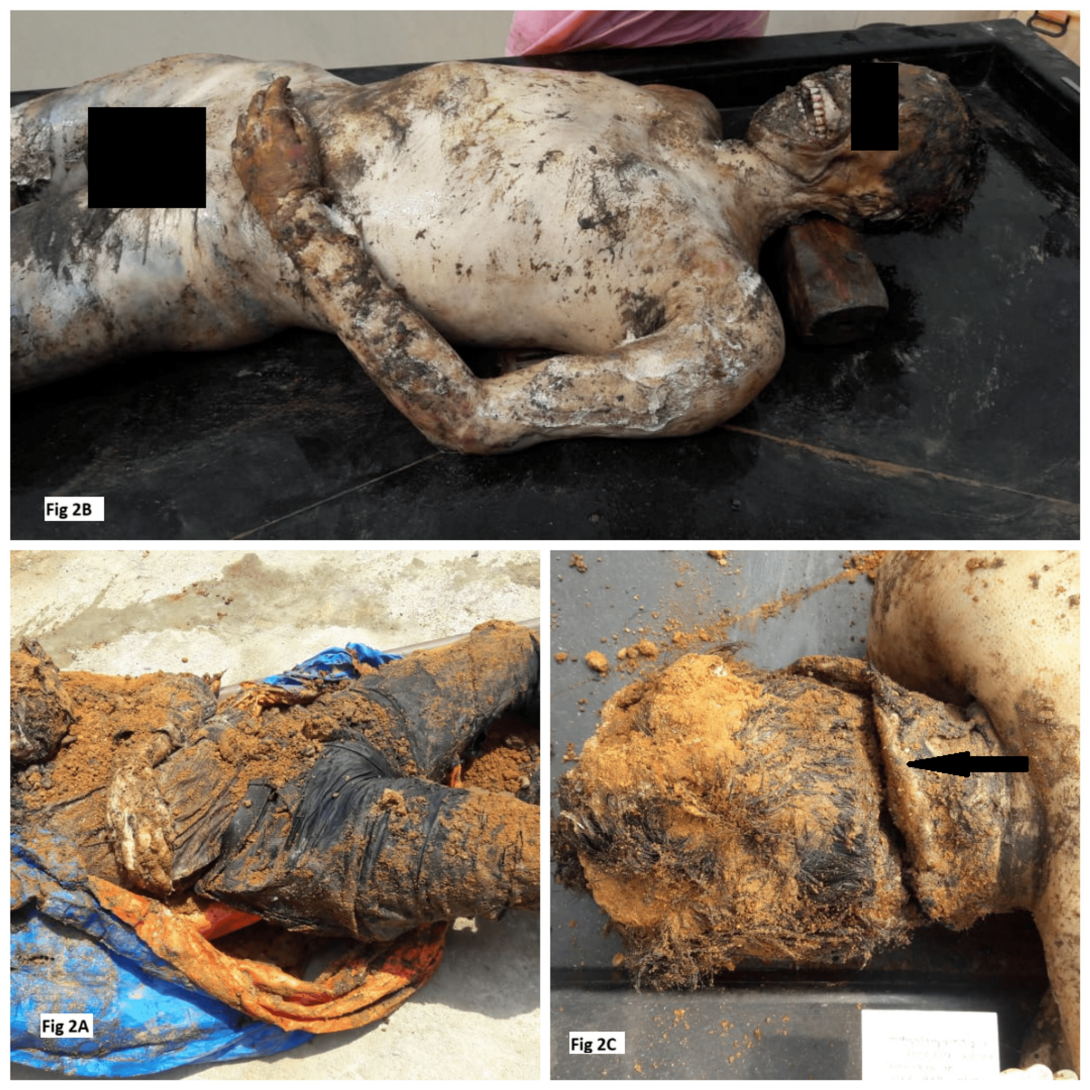
A) The exhumated dead body at the crime scene. B) Adipocere formed all over the body. C) The chop wound and its features are conserved due to adipocere formation.

In this case, the wife of the deceased had complained that her husband had been missing for the past 11 days. During interrogation, the suspect admitted that he had murdered the deceased and buried the body 11 days before. The accused identified the location of the burial, the process of exhumation started in the morning, and the exhumed body was transferred for autopsy at the mortuary of a tertiary care medical center in South India (Figure 2A).

The corpus of an adult male wrapped in a blue and orange tarpaulin sheet with edges showed a burnt black margin. The body was covered with sand and mud, emitting a foul, rancid offensive smell with whitish soft adipocere formation seen all over the body (Figure 2B). Hairs were easily pluckable from the scalp and in the pubic region. Degloving and destocking of the skin were appreciable on both hands and feet. The eyes and nose were soft and greasy. The teeth were loosened and present in the respective sockets. A surgical scar mark was found on the right side of the chest over the right clavicle. On further examination, there was a chop wound over the occipital region, placed obliquely, with complete transection of the spinal cord at the level of first and second cervical vertebra associated with fracture ante-mortem in nature with blood infiltration in bony trabeculae (Figure 2C). The age of the body was estimated to be 40-50 years by the osteological examination. Internal organs showed decomposition changes. Toxicological analysis of viscera ruled out poisoning; however, preservation of the molar tooth was done for DNA fingerprinting and cross-matching. The cause of death has been opined the death due to shock and hemorrhage due to complete transection of the spinal cord resulting from a cut injury to the neck by a moderate to heavy, hard, sharp cutting weapon, and the time since death was 10 to 12 days.

Case 3

**Figure 3:**
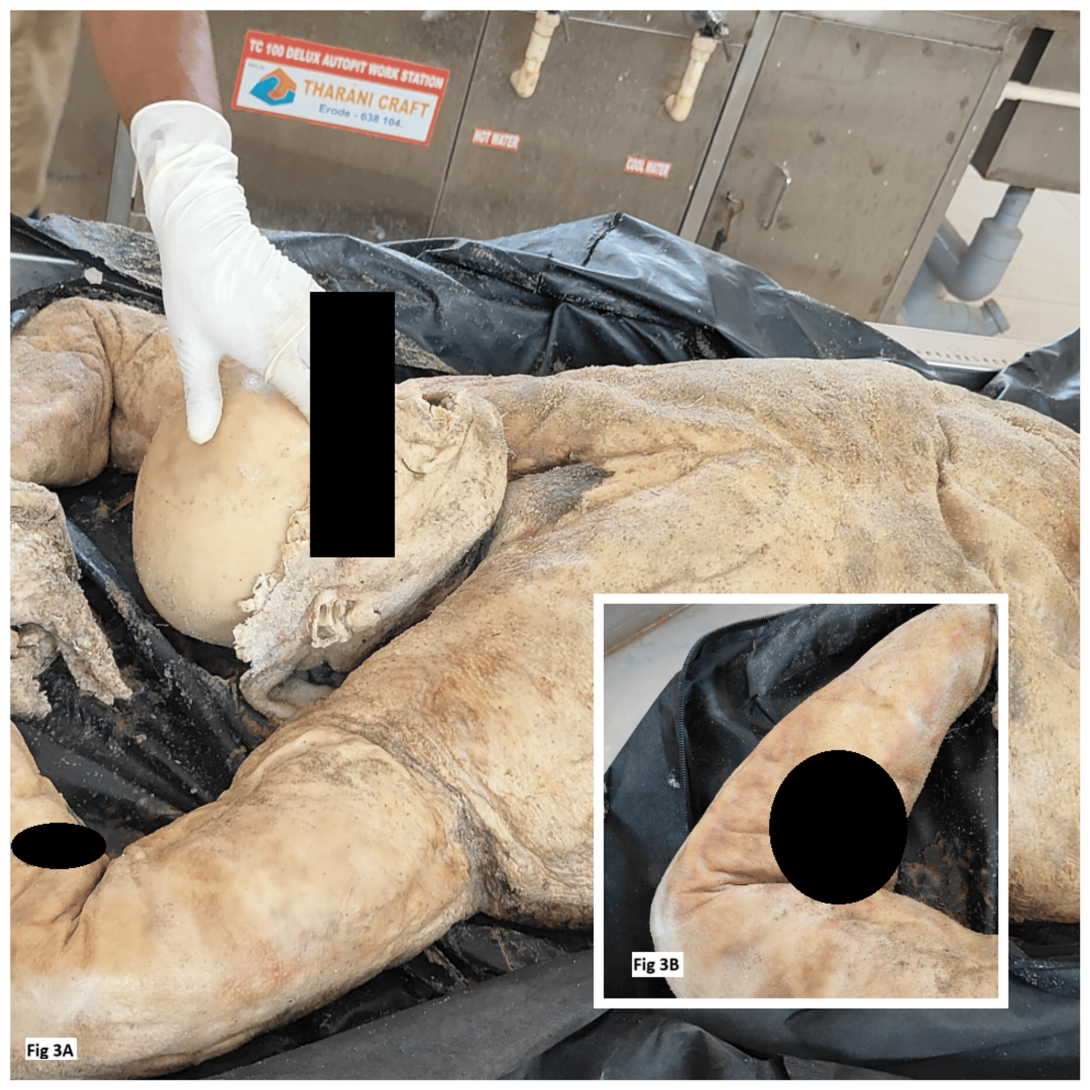
A) The body demonstrating adipocere formation. B) The tattoo mark on the right forearm facilitates the positive identification of the deceased. (The tattoo marks in Figure 3A and 3B were obscured to conceal the identification of the deceased.)

A corpus of an adult male washed ashore on the shoreline of a fishing village in Southern India. The investigating officer registered a case and kept the body in cold storage for identification at a nearby mortuary of the District Headquarters Medical Centre. No missing person case was filed in and around that district. At the time of recovery, the body and face of the deceased were completely disfigured due to the process of adipocere formation (Figure 3A).

However, a tattoo mark of a cross symbol of Christianity was present over the ventral aspect of the forearm (Figure 3B), and a sacred thread was loosely tied around the wrist of the hands. The photograph of the deceased, the tattoo mark, and the sacred thread were sent to other police stations and the police control room for positive identification. After two days, the investigating officer received information that a 50-year-old man was a missing case registered at a police station in Kerala, around 350 kilometers away from where the body was recovered. A group of fishermen ventured out to sea for fishing 10 days ago, and in the midsea, due to high tides and unfavorable weather conditions, the boat capsized and sank. The family members have been contacted through phone, and the deceased was identified by his daughter based on the tattoo mark on his limb and the sacred thread on his wrist.

On examination, the body was naked, wet, and smudged with beach sand and water weeds in places. Yellowish-white, waxy adipocere formation occurred all over the body, and it was emitting a rancid smell. The scalp hairs were missing, and the skin and soft tissues over the skull had lost their attachment, exposing the skull. The face, ears, and nose were deformed. The eyeballs were not found in the orbital cavity. Facial features were not identifiable. Skin and adipose tissues were separated from the underlying body structure and loosely fluctuated over the body framework. The fingers in both hands were missing, and the hands showed gnawing effects. Both feet were destocked and also showed gnawing effects. No external injury was appreciable. All the teeth were missing from their respective sockets. The hyoid bone was intact. Routine viscera analysis ruled out the possibilities of poisoning or other stupefying agents. Preservation of the molar tooth was done for DNA fingerprinting and cross-matching for identification. Sternum and clavicle were preserved and sent for diatom analysis. The cause of death was given as antemortem drowning and its complications, and the time since death was 10 to 14 days.

## Discussion

Adipocere is a yellowish to gray-white waxy postmortem material that forms from the decomposition of adipose tissues within a body, with cheesy and ammonia odor [4]. It is a form of alteration in the decomposition process, where the process of decomposition slows or takes a pause under suitable environmental conditions, and adipocere formation occurs [10]. Previously, it was presumed that adipocere formation occurs only in subcutaneous tissues where fat content is higher. At times, the putrefactive gases formed in the body pressurise the liquefied fat to penetrate the tissues where fat content is low and help in the formation of adipocere. The rate of adipocere formation is not the same in every individual, even if it may even show variations between different parts of the body, mainly depending on the fat content of the region. Furthermore, the variation is evident between males and females, young and old age, probably due to the high fat composition in females as comparative to males and old age people compared to young age people. Bereuter et al., in their work on a man and woman in the same car submerged in a mountain lake, showed that the man was skeletonized, while the woman’s soft tissue was conserved by adipocere [11]. The adipocere starts to form within the subcutaneous tissue, which spreads outward and covers the entire body. The cheek, buttock, anterior abdominal wall, thighs, and breast are the common areas of adipocere formation. At times, the hand and feet may be free of adipocere formation and become skeletonized and disarticulated from the rest of the body.

It is also essential to perceive the facts rather than enhance or favor the adipocere formation as it has the property of inhibiting decomposition and complete degradation if the dead body. Because of this property, adipocere plays a vital role in forensic investigations as it retains the body structures and facial features, facilitates the positive personal identification of the individual, to find out the cause and manner of death by preserving the injuries. In addition, adipocere can aid or hinder the investigating agencies to estimate time since death as the formation occurs at environmental and seasonal conditions, which can be backtracked easily, if the time since death is longer. In such cases, the adipocere formation occurs over a shorter duration and may confuse the investigator in estimating time since death [12,13].

In our first case, the victim was stabbed to death, followed by the perpetrator chopping the dead body into pieces and then packing in a sack and throwing it in a damp yard. The warm, moist, and anaerobic environment favored the adipocere formation. The adipocere covers the entire body and preserves the facial features, and the findings of stab wound, including margins, edges, blood infiltrations in bony trabeculae, and the surrounding soft tissues were easily appreciable and differentiable, which makes the unequivocal interpretation of the wounds. There were antemortem stab wounds and postmortem chopped wounds, which were distinguishable. The time taken for adipocere formation was around two days. Mohan Kumar TS et al. had also documented in their case report that the time taken for adipocere formation was two days. In this case, the medicolegal implications of adipocere played a major role in aiding the investigating agencies. Firstly, the adipocere formed over the face maintained the integrity of facial features, which simplifies the process of identification by the family members of the deceased. Moreover, the features of the stab wound were sustained and useful to find out the cause of death, as it arrests the normal process of putrefaction, which usually disintegrates the features of the injuries, in turn concealing the positive outcomes of the cause of death. In addition, the time since death can be calculated from the adipocere, but the time taken for the adipocere formation varies drastically in different regions of the world.

In the second case, a chop wound over the nape of the neck was identified, and it was preserved due to adipocere formation. A similar case was reported by Gupta M and Jain GV [14], where a female dead body was recovered from the pond, packed in a plastic bag in the winter season with extensive adipocere formation and a cut-throat injury. The cut-throat wound and the internal structures of the neck clearly demonstrated that the injury was caused by a sharp-edged weapon. Furthermore, the body being packed in a plastic bag eliminates the possibility of damage due to predators.

In the third case, because of adipocere formation, the tattoo mark present in the body is well preserved even after days, which helped in the positive identification of an individual. If not for adipocere formation, the features might have been lost, and it would have led to the deceased not being identified, since most countries around the world lack ante-mortem records, which can be used for identification.

The findings from each case highlight the significant role of meticulous examination of adipocere formation in the forensic investigation process. Here, we have discussed cases related to homicide, accidental drowning, and concealed burial. In each of these cases, adipocere formation helped forensic experts to distinguish between ante-mortem and postmortem wounds and also to establish the cause of death with positive identification. The forensic professional with knowledge of adipocere formation can enhance investigation process and ensure justice for affected families.

## Conclusions

The cases that were discussed in this case series help us to understand the forensic significance of adipocere formation in medicolegal investigation. Adipocere formation helps in the preservation of characteristics of a dead body, which is not the case with decomposition; hence, it aids in the identification of an individual along with finding the cause and manner of death. Adipocere formation brings challenges in estimating the time since death for forensic experts, but it also opens an enormous opportunity to study and research about adipocere along with recent advancements for attaining brilliance in postmortem analysis and medicolegal investigation.
